# Supplementary material for: Vision transformer with masked autoencoders for referable diabetic retinopathy classification based on large-size retina image
Source: PLoS One. 2024 Mar 6;19(3):e0299265. doi: 10.1371/journal.pone.0299265 (PMC10917269; doi:10.1371/journal.pone.0299265)
Supplement: Attachment — Submitted filename: Response to Reviews.docx [file pone.0299265.s001.docx]

**Response to Reviews**

**Manuscript ID**: PONE-D-23-29847

**Title**: Vision transformer with masked autoencoders for referable diabetic retinopathy classification based on large-size retina image

We have carefully revised our manuscript according to the journal requirements and comments by editor and reviewers. And we have highlighted the revised text in *blue* type in our revised manuscript. Here is our reply to the journal requirements and review comments.

**Journal requirements:**

1. *When submitting your revision, we need you to address these additional requirements. Please ensure that your manuscript meets PLOS ONE's style requirements, including those for file naming. The PLOS ONE style templates can be found at*

*https://journals.plos.org/plosone/s/file?id=wjVg/PLOSOne_formatting_sample_main_body.pdf and* [*https://journals.plos.org/plosone/s/file?id=ba62/PLOSOne_formatting_sample_title_authors_affiliations.pdf*](https://journals.plos.org/plosone/s/file?id=ba62/PLOSOne_formatting_sample_title_authors_affiliations.pdf)*.*

**Our Reply**: We have formatted and revised our manuscript following the style requirements of PLOS ONE.

2. *Please note that PLOS ONE has specific guidelines on code sharing for submissions in which author-generated code underpins the findings in the manuscript. In these cases, all author generated code must be made available without restrictions upon publication of the work. Please review our guidelines at*

*https://journals.plos.org/plosone/s/materials-and-software-sharing#loc-sharing-code and ensure that your code is shared in a way that follows best practice and facilitates reproducibility and reuse.*

**Our Reply**: We have uploaded our code at: <https://github.com/CNMaxYang/VMLRI>. And we have entered this address in the online submission system.

3. *We note that the grant information you provided in the ‘Funding Information’ and ‘Financial Disclosure’ sections do not match. When you resubmit, please ensure that you provide the correct grant numbers for the awards you received for your study in the ‘Funding Information’ section.*

**Our Reply**: We are sorry for this mistake, and we have deleted the funding information in our revised manuscript according to the journal requirement.

4. *We note that Figure 1 in your submission contain copyrighted images. All PLOS content is published under the Creative Commons Attribution License (CC BY 4.0), which means that the manuscript, images, and Supporting Information files will be freely available online, and any third party is permitted to access, download, copy, distribute, and use these materials in any way, even commercially, with proper attribution. For more information, see our copyright guidelines:* [*http://journals.plos.org/plosone/s/licenses-and-copyright*](http://journals.plos.org/plosone/s/licenses-and-copyright)*. We require you to either (1) present written permission from the copyright holder to publish these figures specifically under the CC BY 4.0 license, or (2) remove the figures from your submission:*

*a. You may seek permission from the original copyright holder of Figure 1 to publish the content specifically under the CC BY 4.0 license. We recommend that you contact the original copyright holder with the Content Permission Form (http://journals.plos.org/plosone/s/file?id=7c09/content-permission-form.pdf) and the following text:“I request permission for the open-access journal PLOS ONE to publish XXX under the Creative Commons Attribution License (CCAL) CC BY 4.0 (http://creativecommons.org/licenses/by/4.0/). Please be aware that this license allows unrestricted use and distribution, even commercially, by third parties. Please reply and provide explicit written permission to publish XXX under a CC BY license and complete the attached form.” Please upload the completed Content Permission Form or other proof of granted permissions as an ""Other"" file with your submission. In the figure caption of the copyrighted figure, please include the following text: “Reprinted from [ref] under a CC BY license, with permission from [name of publisher], original copyright [original copyright year].”*

*b. If you are unable to obtain permission from the original copyright holder to publish these figures under the CC BY 4.0 license or if the copyright holder’s requirements are incompatible with the CC BY 4.0 license, please either i) remove the figure or ii) supply a replacement figure that complies with the CC BY 4.0 license. Please check copyright information on all replacement figures and update the figure caption with source information. If applicable, please specify in the figure caption text when a figure is similar but not identical to the original image and is therefore for illustrative purposes only.*

**Our Reply**: We feel very sorry for this copyright issue. Figure 1 is a concept graph of our proposed method. Thus, we have redesigned figure 1 to avoid using copyrighted graph.

**Additional Editor Comments:**

*In conclusion, after careful review and consideration of the paper titled "Vision transformer with masked autoencoders for referable diabetic retinopathy classification based on large-size retina image", the initial decision is major revision. This decision is based on the consensus of the reviewers and the following concerns and contributions of the paper:*

*From R1, the authors should compare the method with other state-of-the-art CNN models, state the novelty and contribution of the proposed work, the limitations of the method, and other details in implementation and result analysis.*

*From R2, the authors should make the architecture clear. R2 also mentions some issues in the writing and layout in tables, contributions and figures.*

**Our Reply**:

We are very grateful to the editor for giving us the opportunity to revise our manuscript.

According to the professional and useful comments by Reviewer #1, we have added a separated paragraph at the end of “Experiment and results” section to compare the present network with state-of-the-art methods. And we have also revised our discussion part to illustrate the contribution and limitation of the proposed method. Please see lines 267-290 on pages 15-17, lines 304-310 and 313-316 on pages 17-18.

Based on the comments and suggestion by Reviewer #2, we have revised the figures and tables in our manuscript. We have redesigned figure 1 and described the details in this figure. And the hyperparameters were summarized in table 3. Please see lines 102-116 on pages 5-6, lines 156-161 on page 8 and line 196 on page 11.

**Reviewer #1:**

*The author implemented the article entitled "Vision transformer with masked autoencoders for referable diabetic retinopathy classification based on large-size retina image". There are many queries need to be addressed.*

**Our Reply**: We appreciate the valuable and useful comments and suggestions by Reviewer #1. We have revised our manuscript according to the following review comments.

1. *Did the authors compared the Vision transformer performance with any other state of art CNN?*

**Our Reply:** Thank you very much for your comment. We have added a separated paragraph at the end of “Experiment and results” section to compare the present network with state-of-the-art methods. In order to make this clear, we have also added Table 9 to show the accuracy, AUC, sensitivity and specificity by the present method and other state-of-the-art methods on the same dataset. And the results show that the accuracy of the proposed VMLRI is very close to that by InceptionV3, while the AUC and specificity are highest. Please see lines 267-290 on pages 15-17.

2. *Since already many researchers implemented Vision Transformer in Diabetic retinopathy database. Then what is the novelty of the proposed work?*

**Our Reply:** We agree with this comment that Vision Transformer (ViT) has been used by many researchers in diabetic retinopathy (DR). However, because of lack of inductive bias, ViT may not perform better than CNN when the training data is limited. This issue is encountered in the field of DR because the cost of acquiring and annotating medical images is high. Currently, the pre-trained weights of ViT on ImageNet are generally used to improve the performance of ViT. However, the input image size in this kind of method should be restricted to 224×224, and the model structure of ViT is difficult to change because training a modified ViT on tens of millions of natural images is quite challenging. Therefore, a new method named VMLRI was developed in the present work. We pre-trained ViT on the retinal images larger than 224×224 using MAE, and compared the classification results with ViT pre-trained with ImageNet1k. The results indicate that the improvement of the present model is superior to that pre-trained with ImageNet. The accuracy, AUC, highest sensitivity and highest specificity of the present model are 93.42%, 0.9853, 0.973 and 0.9539, respectively. This study shows that MAE can provide more flexibility to the input image and substantially reduce the number of images required. Meanwhile, the pretraining dataset scale in this study is much smaller than ImageNet, and the pre-trained weights from ImageNet are not required also.

In order to make the novelty of the present work clear, we have revised the “Experiment and results” and “Discussion” sections. Please see lines 224-225 and lines 226-228 on pages 12-13, lines 232-237 on page 13, lines 256-257 on page 15 and lines 267-290 on pages 15-17.

3. *Literature review given in the related work is not sufficient. Need to add some more recent papers*.

**Our Reply:** Thank you for your suggestion. We have added up-to-date literatures in our revised manuscript. Please see references 29-31 and lines 82-91 on pages 4-5.

4. *Training and testing data spilt?*

**Our Reply:** The data was divided into two parts: part 1 was used for pre-training, and the other part of data was used for fine-tuning and testing. The pre-training dataset (part 1) includes images from APTOS without official labels, EyePACS, Messidor-2, and OIA-DDR. The dataset for fine-tuning and testing (part 2) consists of images from APTOS with official labels. There is no overlap between the images used for pre-training and those used for fine-tuning and testing. In fine-tuning and testing, the APTOS with official labels was splitted into two classes based on the labels, normal and referable diabetic retinopathy. Subsequently, 10% of images were randomly selected from each class to form the test set including 365 images. Then, 80% and 20% of the remaining 3297 images were used for fine-tuning and validating, respectively.

5. *Whether 5 fold or 10 fold validation carried out?*

**Our Reply:** It is believed that splitting APTOS dataset into training and validation as well as testing set is sufficient for binary classification. Since K-fold cross-validation involves dividing the original data into K non-overlapping subsets and performing K iterations, the K-fold cross-validation was not carried out in the present work.

6. *Information about the hyperparameters used.*

**Our Reply:** We have added Table 3 to summarize the used hyperparameters according to this valuable suggestion. Please see line 196 on page 11.

7. *Have you performed error analysis?*

**Our Reply:** Actually, we did not perform error analysis in the present work. The main reason is that analyzing and improving on misclassified samples of retinal images may degrade the performance of models using ImageNet weights. Thus, in order to compare the impact of retinal image weights and ImageNet weights obtained using MAE pre-training on ViT performance, the error analysis was not performed in the present work. Similar strategy was adopted by a few researchers [43-46].

[43] Zhang G, Sun B, Zhang Z, Pan J, Yang W, Liu Y. Multi-model domain adaptation for diabetic retinopathy classification. Frontiers in Physiology. 2022;13:918929.

[44] Zhang C, Lei T, Chen P. Diabetic retinopathy grading by a source-free transfer learning approach. Biomedical Signal Processing and Control. 2022;73:103423.

[45] Vives-Boix V, Ruiz-Fernández D. Diabetic retinopathy detection through convolutional neural networks with synaptic metaplasticity. Computer Methods and Programs in Biomedicine. 2021;206:106094.

[46] Jaskari J, Sahlsten J, Damoulas T, Knoblauch J, Särkkä S, Kärkkäinen L, et al. Uncertainty-aware deep learning methods for robust diabetic retinopathy classification. IEEE Access. 2022;10:76669-81.

8. *Methods need to be elaborated.*

**Our Reply:** Thank you very much for your comment. We have redrawn Figure 1 and added detailed explanation on the proposed method. Please see lines 102-116 on pages 5-6.

9. *Discussion is incomplete. The author should discuss the results of their proposed work with existing related literature works.*

**Our Reply:** Thank you for your comment. We have added a separated paragraph at the end of “Experiment and results” section to compare the present network with state-of-the-art methods. And we have also revised our discussion part. Please see lines 267-290 on pages 15-17 and lines 308-310 on page 18.

10. *Include the limitations and future scope of the work.*

**Our Reply:** We have added a short discussion on the limitations and future scope of the present work. Please see lines 304-310 on pages 17-18 and 313-316 on page 18.

11. *Result analysis need to be improved in Result section.*

**Our Reply:** Thank you for your comment. We have rewritten the content in the “Results and analysis” section. And we have also added a separated part to compare the present network with state-of-the-art methods. Please see line 224-228 on pages 12-13, lines 232-237, on page13 and lines 256-257 on page 15.

**Reviewer #2:**

1. *while talking about datasets: start with the APTOS dataset as this was the one mentioned rigorously within the introduction, then move forward to the other datasets.*

**Our Reply:** Thank you for your suggestion. We have adjusted the order of the dataset and firstly introduced APTOS dataset in "Datasets" section. Please see lines 125-128 on page 7.

2. *In the introduction: explain what DeiT-S and other techniques stand for as you added the details for CNN and ViT.*

**Our Reply:** Thanks for your comments, we have added explanation for DeiT-S in introduction. Please see lines 50-53 on page 3.

3. *Confusion between the closing paragraphs within the introduction and the related work. why dont you move all this part to be combined with the related work. Divide the related work to work conducted on standard size retinal images and larger retina images in order to present the drawbacks mentioned within the introduction based on solid facts.*

**Our Reply:** Thanks for your comment. We have moved the closing paragraphs in the introduction to the end of "Related work" section. However, to our knowledge, most of available work use standard size retinal images for pre-training. It is lack of work related to Transformer on large size retinal images. Please see lines 117-122 on page 6.

4. *Figures needs more labeling between arrows in order to better understand the sequence.*

**Our Reply:** Thank you very much for your comment. We have redrawn Figure 1 and provided an explanation to the methods shown in Figures 1 and 2. Please see lines 102-116 on pages 5-6 and lines 156-161 on page 8.

5. *Move the contributions to the conclusions, replace those with objectives instead to enhance the readability.*

**Our Reply:** Thank you for your comment. We have rewritten the final part of "Related work" according to the suggestion. Please see lines 117-122 on page 6.

6. *For the results tables, highlight the best performing techniques through all datasets and all evaluation criterea*.

**Our Reply:** Thank you for your comment. We have indicated the maximum values for each evaluation metric with bold fonts in Tables 6-9. Please see line 238 on page 13, line 244 and line 248 on page 14 and lines 279-280 on page 16.

7. *The architecture is very unclear, every component needs to be explained separately with the motivation behind it and what it adds to the overall technique*.

**Our Reply:** Thank you for your comment. We have redrawn the architecture diagram, added a legend, and provided detailed descriptions on the architecture of figures 1 and 2. Please see lines 102-122 on pages 5-6 and lines 156-161 on page 8.

We would like to take this opportunity to again express our great appreciation to the valuable and useful comments and suggestions by the editor and reviewers. We hope we have revised our manuscript in a satisfactory way and it can be considered for possible publication in **PLOS ONE**.

Yours sincerely,

**Peng XU** *Ph.D., Professor*

College of Science, China Jiliang University

**Add.**: 258 Xueyuan Street, Hangzhou 310018, P.R. China

**Tel.**: 86-571-86875632 (O), 86-13868125229 (H)

**Email**: xupeng@cjlu.edu.cn

**ORCiD**: orcid.org/0000-0002-4349-5627
