# Supplementary material for: Vision transformer with masked autoencoders for referable diabetic retinopathy classification based on large-size retina image
Source: PLoS One. 2024 Mar 6;19(3):e0299265. doi: 10.1371/journal.pone.0299265 (PMC10917269; doi:10.1371/journal.pone.0299265)
Supplement: Attachment — Submitted filename: Response to Reviewers.docx [file pone.0299265.s002.docx]

**Response to Reviews**

**Manuscript ID**: PONE-D-23-29847R1

**Title**: Vision transformer with masked autoencoders for referable diabetic retinopathy classification based on large-size retina image

We have carefully revised our manuscript according to the journal requirements and comments by editor and reviewers. And we have highlighted the revised text in *red* type in our revised manuscript. Here is our reply to the journal requirements and review comments.

**Journal requirements:**

*Please review your reference list to ensure that it is complete and correct. If you have cited papers that have been retracted, please include the rationale for doing so in the manuscript text, or remove these references and replace them with relevant current references. Any changes to the reference list should be mentioned in the rebuttal letter that accompanies your revised manuscript. If you need to cite a retracted article, indicate the article’s retracted status in the References list and also include a citation and full reference for the retraction notice.*

**Our Reply**: We have checked our reference list, and we ensure we didn't cite any retracted paper.

**Additional Editor Comments:**

*Please address the minor comments on the diagram, logic flow and literature reviews. After the modification, the manuscript should be ready to publish.*

**Our Reply**: We appreciate the comments from the reviewers. We have revised Figures 1 and 2 by adding annotations to make them clear. Additionally, we have revised the related work section, and added subsections to improve readability.

**Comments by Reviewer #1:**

*The authors have addressed the reviewer comments. Hence the article can be accepted in its current form.*

**Our Reply**: We appreciate the positive comments by Reviewer #1.

**Comments by Reviewer #2:**

*Most of the comments have been covered (greatest thanks for your effort), however I will reiterate two comments that will make the paper up to standard:*

**Our Reply**: We appreciate the valuable and useful comments and suggestions by Reviewer #2. We have revised our manuscript according to the comments. Please see the text in lines 75-110 and Figures 1 and 2.

1. *Diagrams to be clearer (especially ViT diagram), The logic flow is very unclear without reading the text.*

**Our Reply:** Thank you for this valuable suggestion. We have added the annotations in Figures 1 and 2 to improve the readability. Please see line 130 on page 7, and line 171 on page 9.

2. *Dividing the literature review into sub sections based on the size of the dataset worked on.*

**Our Reply:** Thanks for your useful comments. In the related work section, we divided this section into two parts: Transformer and MAE. Only the DR retinal images were focused on in Transformer subsection. To our knowledge, the applications of MAE in DR classification are lesser. Thus, various 2D and 3D medical images with different target diseases and imaging modalities were included in MAE subsection. Therefore, it is difficult to categorize the literature review directly based on the size of dataset. However, in order to highlight the motivation of using higher-resolution medical images proposed in this paper, we have included the details of image resolution for each dataset in the literature review. Please see the revised text in lines 75-110.

We would like to take this opportunity to again express our great appreciation to the valuable and useful comments and suggestions by the editor and reviewers. We hope we have revised our manuscript in a satisfactory way and it can be considered for possible publication in **PLOS ONE**.

Yours sincerely,

**Peng XU** *Ph.D., Professor*

College of Science, China Jiliang University

**Add.**: 258 Xueyuan Street, Hangzhou 310018, P.R. China

**Tel.**: 86-571-86875632 (O), 86-13868125229 (H)

**Email**: xupeng@cjlu.edu.cn

**ORCiD**: orcid.org/0000-0002-4349-5627
